# Exploration of Semantic Label Decomposition and Dataset Size in Semantic Indoor Scenes Synthesis via Optimized Residual Generative Adversarial Networks

**DOI:** 10.3390/s22218306

**Published:** 2022-10-29

**Authors:** Hatem Ibrahem, Ahmed Salem, Hyun-Soo Kang

**Affiliations:** 1 Department of Information and Communication Engineering, School of Electrical and Computer Engineering, Chungbuk National University, Cheongju-si 28644, Korea; 2 Electrical Engineering Department, Faculty of Engineering, Assiut University, Assiut 71515, Egypt

**Keywords:** generative adversarial networks, convolutional neural networks, image-to-image translation, semantic image synthesis

## Abstract

In this paper, we revisit the paired image-to-image translation using the conditional generative adversarial network, the so-called “Pix2Pix”, and propose efficient optimization techniques for the architecture and the training method to maximize the architecture’s performance to boost the realism of the generated images. We propose a generative adversarial network-based technique to create new artificial indoor scenes using a user-defined semantic segmentation map as an input to define the location, shape, and category of each object in the scene, exactly similar to Pix2Pix. We train different residual connections-based architectures of the generator and discriminator on the NYU depth-v2 dataset and a selected indoor subset from the ADE20K dataset, showing that the proposed models have fewer parameters, less computational complexity, and can generate better quality images than the state of the art methods following the same technique to generate realistic indoor images. We also prove that using extra specific labels and more training samples increases the quality of the generated images; however, the proposed residual connections-based models can learn better from small datasets (i.e., NYU depth-v2) and can improve the realism of the generated images in training on bigger datasets (i.e., ADE20K indoor subset) in comparison to Pix2Pix. The proposed method achieves an LPIPS value of 0.505 and an FID value of 81.067, generating better quality images than that produced by Pix2Pix and other recent paired Image-to-image translation methods and outperforming them in terms of LPIPS and FID.

## 1. Introduction

The recent development in unsupervised techniques has shown generative adversarial networks’ (GAN’s) great ability to generate new images that highly imitate real-world images. GAN was proposed by Goodfellow et al. [1] as a new framework of generative models that can learn via an adversarial technique. The paired image-to-Image translation has recently drawn the attention of computer vision researchers as a difficult task that has been eased using GAN. Unlike the conventional generative models [2,3], which generate low-quality images, GAN can produce high-quality images following the same training data distribution as GAN learns the distribution of the data starting with a normally distributed noise. Many GAN-based methods have been developed recently to solve hundreds of computer vision problems. Many recent GAN models suggested using a conditional input image to generate another output image of the desired application. The most popular is Isola et al. [4]. They proposed Pix2Pix GAN, which allows image-to-image translation for different applications, such as labels to street scene, labels to façade, grayscale to a color image, day to night, and drawing to a realistic image. Their proposal [4] adopted U-Net [5] architecture, which was originally proposed for medical image semantic segmentation. In this paper, we propose a modified GAN model to perform an image-to-image translation with higher quality than the mentioned study [4] and yet far less complexity. In this paper, we modify, optimize, and recreate the Pix2Pix GAN model with various architectures and techniques to generate high-quality images, specifically indoor images, which are beneficial in interior design, architectural renovations and remodeling, VR/AR applications, and image style transfer. The contribution of our study can be briefly stated as follows:

- We propose three different UNet-like architectures for the generator network and three different discriminator architectures to fit with each generator architecture. We compare them and highlight the advantages and disadvantages of each architecture.
- In the architecture of the generator, we compare two merging techniques (concatenation and addition) of the same feature size blocks in the encoder and the decoder. We show that merging using addition gives the same, if not better, results than merging features using concatenation, while the addition operation is less complex than concatenation.
- We propose relatively lightweight encoder-decoder GAN models with residual connections, less computational complexity, and higher quality of the generated images than the original U-Net, which is the core of the image-to-image translation approach using GAN (Pix2Pix).
- We explore the effect of the number of class labels (including the same label decomposition) and the number of training samples on the quality of the generated artificial images on the NYU depthV2 dataset and ADE20K Indoor subset.

**Figure 1:**
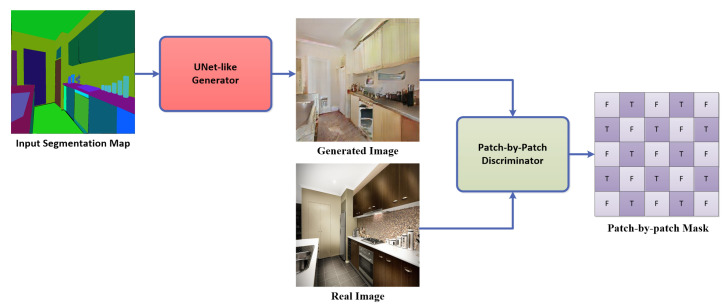
Overview of the proposed paired Image-to-Image translation technique.

An overview of the paired Image-to-Image translation using Pix2Pix [4] is shown in Figure 1. A UNet-like generator architecture generates an artificial image, and a discriminator architecture (patch-by-patch classifier) is used to discriminate between the artificial and real images. Along the training process, as the discriminator becomes better and better, the generator also becomes better accordingly. The formation of this paper is as follows. First, we discuss the related work in Section 2. We present the proposed work in Section 3, the benchmarks and experiment preparation in Section 4, and the experiments we did and the obtained results in Section 5. Finally, we state the future work and conclusions of our study in Section 6 and Section 7, respectively. The explanation of all undefined abbreviations used throughout the paper exists in Abbreviations.

## 2. Related Work

GAN [1] mainly consists of two main networks, a generator network and a discriminator network. The generator network aims to generate images from the input data (a specific probability distribution or conditional known data); however, the discriminator network aims to recognize whether the generated image is real or fake. The training of both networks is done adversarially in which the more the generator gets better, the more the discriminator gets better. The objective function of GAN is as stated in Equation (1):

(1) $$L_{GAN}=E_{x∼P(x)}\left[log\left(D\left(x\right)\right)\right]+E_{z∼P(z)}\left[log\left(1-D\left(G\left(z\right)\right)\right)\right]$$

where *z* represents the input vector fed to the generator, *P*(*x*) is the real data distribution, and *p*(*z*) is the input noise distribution. *D*(*x*) is the discriminator’s estimate of the real data probability prediction, and *G*(*z*) is the generator output. According to this equation, the generator aims to minimize this function and the discriminator aims to maximize it. The recent GAN-based approaches depend mainly on deep convolutional neural networks (DCNN) to generate images. DCGAN [6] was one of the first approaches to use DCNN to generate realistic, complex images; they fed a 100-dimensional uniform distribution vector to an up-sampling CNN architecture, and the generated image was fed to another CNN discriminator network to classify the image to detect whether it is a real or a fake image. However, DCGAN does not offer the ability to control the attributes of the output image. Mirza et al. proposed conditional GAN (CGAN), in which they offered to generate images conditioned on the class labels encoded in the input vector, which is fed to the generator instead of the uniform distributed noise vector. This approach helped in developing many GAN variants for different tasks and applications, such as CycleGAN [7], styleGAN [8], and Pix2Pix [4]. Isola et al. [4], who proposed Pix2Pix, adopted CGAN and DCGAN concepts. They developed the image-to-image translation with a conditional input to translate one image from a specific domain to a relevant image in another domain, which was the key for the previously mentioned applications. They employed U-Net as the generator network to construct the fake images and used a CNN discriminator (PatchGAN [4]) to recognize real/fake images using a patch-by-patch classification approach, where the output from the discriminator was a 30 × 30 map.

Recent research on semantic image synthesis using GAN improved the quality of the generated images using more complex pipelines. Wang et al. [9] proposed “Pix2PixHD”, a high-resolution image synthesis with conditional GANs, for realistic image synthesis from the semantic labels using a multiscale generator and discriminator; they first trained a residual generator on lower resolution images, then another residual generator to generate the final high-resolution image. They also incorporated object instance segmentation information to improve the generated image’s quality. Park et al. [10] proposed a semantic image synthesis technique using a spatially-adaptive normalization layer (SPADE), in which they replaced the batch normalization layers with a new spatially adaptive layer to modulate the activations in the normalization layer; they demonstrated that the batch normalization causes semantic information loss of the input features. Tan et al. [11] also proposed a semantic image synthesis technique via modeling of the semantic class distribution; they could achieve competitive results by class parameters modulation as continuous probability distributions instead of discrete distributions. They also proposed a noise-remapping technique to facilitate the training process. Sushko et al. [12] proposed an adversarial supervision-based model to generate high-quality generated images by designing the discriminator as a semantic segmentation network. They strengthened the discriminator through semantic and spatial awareness, which is also reflected in the generator. They also employed the perceptual loss [13] to achieve their excellent results. Wang et al. [14] recently proposed a denoising diffusion probabilistic model for image synthesis. They fed a noisy image to the encoder of a U-Net generator and fed the semantic layout to the decoder via the SPADE layer instead of directly feeding the semantic layout to the encoder. This technique led to competitive results compared to the previous methods. Although the previously mentioned methods achieved high quality and excellent photo-realistic results, their architectures were complex and slow in training and testing. In this paper, we chose to keep the architectures simple by adopting simple discriminator and generator architectures. while we employed some tricks to improve the quality. We also explore the problems of a simple image synthesis architecture (i.e., Pix2Pix) and solve those issues by optimization and modification of that architecture in order to get high-quality realistic images similar to those of the recent state-of-the-art (SOTA) methods.

## 3. Proposed Method

We modify the original implementation of Pix2Pix [4], which employs a U-Net [5]-like generator architecture and PatchGAN discriminator. First, we propose two other generator architectures employing ResNet [15] and Xception [16] in a shape much similar to U-Net, but we make use of the feed-forward skip connections that improve the training process and prevent overfitting. Second, we compare two merging techniques (addition versus concatenation) in the skip connections that connect the corresponding blocks in the encoder and decoder. We discuss the generator and discriminator architectures in detail in the following subsections.

### 3.1. Proposed Architectures

**Figure 2:**
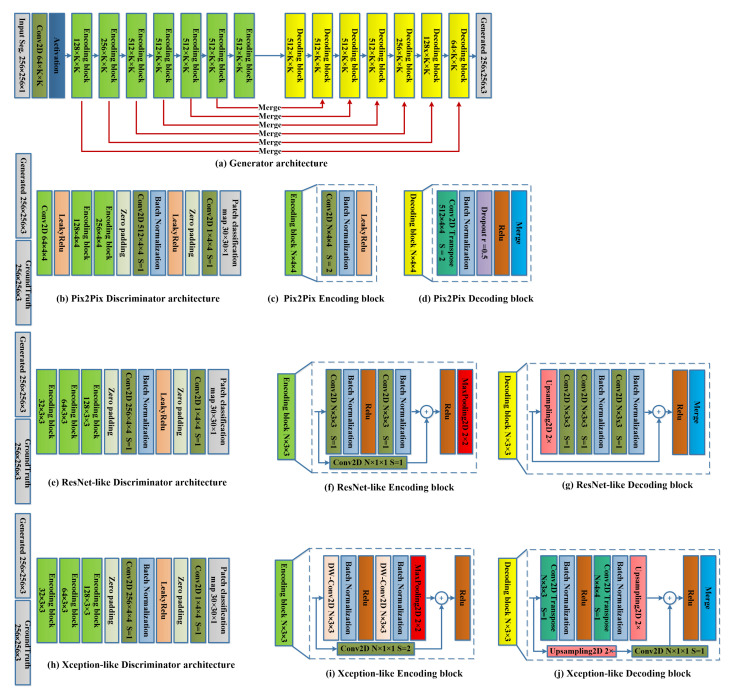
The proposed architectures of generators and discriminators.

We modify the original implementation of Pix2Pix [4], which employs a U-Net [5]-like generator architecture and PatchGAN discriminator. First, we propose two other generator architectures employing ResNet [15] and Xception [16] in shape much similar to U-Net, but we make use of the feed-forward skip connections that improve the training process and prevent overfitting. Second, we compare two merging techniques (addition versus concatenation) in the skip connections that connect the corresponding blocks in the encoder and decoder. We discuss the generator and discriminator architectures in detail for the original Pix2Pix, which uses VGG-based [17] U-Net and VGG-like discriminator architecture. The feature merging by concatenation is used to connect the corresponding blocks in the encoder and decoder. We modified the generator architecture by replacing the concatenation layers with Addition layers to add features in different levels of the encoder and the decoder. Merging by addition is much faster and requires fewer learnable parameters than the case of using the concatenation layer. Figure 2a shows the general generator architecture, an encoder-decoder CNN architecture with skip connections between the encoder and the decoder blocks. The encoder in the generator architecture compresses the input image into a tiny representation, then the decoder stage reconstructs the output image using the small representation and the merging of the different scale features from all the different stages in the encoder to the decoder’s features. The Pix2Pix encoding block, as shown in Figure 2c, consists of a convolutional layer with 4 × 4 kernel size and N number of the filters, where N increases as the depth of the encoder increases (64→128→256→512→512→512→512→512). Each convolutional layer is followed by a batch normalization (BN) layer then leaky-Relu activation. The Pix2Pix decoding block, shown in Figure 2d, is exactly the opposite of the encoder architecture, with the number of channels N going in the opposite sequence to the encoder’s sequence. It employs Conv2D-Transpose with a stride of 2 to up-sample the features followed by batch normalization, a dropout with a rate of 0.5, Relu, and the merging layer. The Pix2Pix discriminator architecture (Figure 2b) is much similar to the encoder part of the generator. A patch-by-patch binary classification layer with sigmoid activation is added at the end of the discriminator to decide whether each region in the generated image is fake or real. The discriminator architecture classifies the image patch-by-patch into a 32 × 32 mask; each pixel in the mask corresponds to a small region of 8 × 8 lowing subsections.

The second implementation, a ResNet-Like Generator, and the discriminator are similar to the original Pix2Pix, but the encoding block is replaced with a custom Residual block (Conv2D→BN→Relu→Conv2D→BN), where the Conv2D layers are 3×3×N. The residual block also contains a residual connection with a 1×1×1 convolution followed by relu and Max-pooling layers, as shown in Figure 2f. The ResNet decoding block, shown in Figure 2g, is an up-sampling (US) residual block consisting of a 2x bilinear-US layer, followed by two Conv2D layers, BN, Conv2D, BN, a skip connection, Relu, and finally the merging layer. Those ResNet-like blocks replace the encoding and decoding blocks in the generator architecture in Figure 2a in the case of the ResNet-like implementation. The ResNet-like discriminator is similar to the Pix2Pix discriminator, but the encoding blocks are replaced with the ResNet-like encoding block, as shown in Figure 2e.

The third implementation is an Xception-like [16] implementation. In this implementation, we adopt the Xception block [16], which mainly uses the Depth-wise separable convolution (DW-Conv2D). The DW-Conv2D consists of a depth-wise convolution (a convolution applied to each channel separately) followed by a point-wise convolution (a 1×1 convolution that projects the input features into the required number of the output features). The DW-Conv2D performance is similar to the convolution but much more efficient in speed and learning performance. The Xception-like encoding block is similar to that of the ResNet-like block, but the Conv2D is replaced with the DW-Conv2D, the max-pooling is moved to be before the residual connection, and the final Relu activation is after it, as shown in Figure 2i. The Xception-like decoding block consists of a repeated Conv2D, BN. Relu, followed by a 2X bilinear-US layer, a residual connection with another 2X bilinear-US and a 1×1×N Conv2D, is adopted, and the residual connection is followed by Relu activation and the merging layer, as shown in Figure 2j. The Xception-like discriminator is similar to the Pix2Pix discriminator while replacing the encoding block with the Xception-like encoding block.

In each of the previously mentioned architectures, we compared the feature merging of the encoder and decoder (using skip connections) using concatenation and addition separately. During training and validation, we used an input image size of 256 × 256 × 1 for the generator input as we fed the semantic input map as a grayscale image in the case of NYU depth V2 [18] since the number of labels was small (14 label or 41 labels), which could be encoded in a grayscale image, which is different from the implementation of pix2pix [4], in which the segmentation image feeds an RGB image with a unique color for each label. In contrast, we fed the semantic input map with a shape of 256×256×3, as the number of labels was large (100 labels) in the case of ADE20K indoor dataset [19].

### 3.2. Loss Function

The loss function for the proposed method was much similar to that for Pix2Pix, using a mixture of GAN loss and L1 loss. The GAN loss can be used individually to learn the image generation process; however, the generated image has a lot of artifacts. The L1 loss individually generates blurry images with much lesser details, so the mixture between them gives the best results according to [4]. The conditioned GAN loss can be defined as stated in Equation (2):

(2) $$L_{GAN}=E_{x,y}\left[log\left(D\left(x,y\right)\right)\right]+E_{x,z}\left[log\left(1-D\left(x,G\left(x,z\right)\right)\right)\right]$$

where D(x,y) is the probability that the generated image is real given the conditioned input (the semantic mask *x* and target real image *y*) and G(x,z) is the generator output given the conditioned input semantic image *x* and noise *z*. The generator (*G*) tries to minimize the loss and the discriminator (*D*) tries to maximize the loss. The *L*1 loss can be defined as stated in Equation (3):

(3) $$L_{L1}=E_{x,y,z}[|y-G\left(x,z\right)|]$$

which tries to minimize the mean absolute error (*L*1 distance) between the generated fake image G(x,z) and the target real image *y*. The generator objective is minimizing the weighted sum of the two objectives $L_{GAN}$ and $L_{L1}$, and it can be stated as shown in Equation (4):

(4) $$GEN_{obj}=argmin_{G}\left(L_{GAN}+\lambda L_{L1}\right)$$

where the value of λ is chosen empirically as 100. The discriminator objective can be stated as shown in Equation (5):

(5) $$DISC_{obj}=argmax_{D}\left(L_{GAN}\right)$$

where the discriminator aims to maximize the GAN loss ($L_{GAN}$) to increase its ability to recognize the fake images generated by the generator.

## 4. Benchmarks and Experiment Preparation

In this section, we present the benchmarks employed in our experiments (NYU depthV2 [18] and ADE20K-indoor [19] datasets), training and test configurations (Section 4.3), and the evaluation metrics (Section 4.4).

### 4.1. NYU Depth-V2

NYU depthV2 is a popular indoor scene dataset for image segmentation and depth estimation. It comprises 1449 semantic segmentation annotated images of 640 × 480 size and contains 14 main and 41 extended labels. The labels contain most objects that can be seen in general indoor scenes, such as bedrooms, kitchens, living rooms, offices, libraries, and bathrooms. We split the dataset into 1000 images for training and 449 images for validation.

### 4.2. ADE20K Indoor Subset

ADE20K is a big dataset for scene parsing; it includes 150 main classes of general outdoor and indoor objects. We selected an indoor scenes (bedrooms, kitchens, living rooms, and toilets) subset called “Home and Hotels” in the main ADE20K dataset as we focused this research on the indoor scenes containing 100 different labels. The subset contained 5991 training images and 517 validation images. The images had variable sizes and were resized to a fixed size of 256×256 during training and validation.

### 4.3. Training and Test Configurations

In the training of the proposed GAN method, we resized the input segmentation masks and the ground truth RGB images into the size of 256×256 to speed up the training process. We used a desktop machine with Nvidia RTX3090 GPU, Intel Core i7-8700 CPU, and 64 Gigabyte RAM. We used the same specification in the test process.

### 4.4. Evaluation Metrcis

We evaluate the proposed GAN models using two popular evaluation metrics in this study. The first evaluation metric was LPIPS [13], which is a perceptual similarity comparison metric that compares the features extracted by a classification CNN, i.e., AlexNet [20], pre-trained on Imagenet [21] for the two images in comparison by calculating the mean squared error (MSE) of the features extracted from last convolutional layer in AlexNet, as shown in Equation (6). The more the LPIPS values, the more diverse the image:

(6) $$LPIPS=\sum_{l}\frac{1}{W_{l}H_{l}}||Alex\_features\left(y\right)-Alex\_features\left(\hat{y}\right){\left|\right|}^{2}$$

where $W_{l}$ and $H_{l}$ are the feature maps’ height and width, respectively. *l* is an iterator over the features count. *y* and $\hat{y}$ are the ground truth and the generated images, respectively.

The second metric is Frechet Inception Distance (FID) [22], which employs the Inception-V3 [23] pre-trained model to extract the last 1D vector generated by the global average pooling layer before the classification layer. Multivariate Gaussian distribution is applied to the 1D vectors to obtain the mean and the covariance; then, FID is measured by calculating the Wasserstein-2 distance between the probability distribution of the real image and the probability distribution of the generated image. Equation (7) represents the FID metric.

(7) $$FID=\left|\right|\mu_{1}-\mu_{2}{\left|\right|}^{2}-Tr\left(C_{1}+C_{2}-2\sqrt{C_{1}C_{2}}\right)$$

where μ and *C* are the mean and covariance matrices. Tr represents the sum of the main diagonal elements in the matrix. The higher the FID, the less probabilistically similar to the real image it is, which means less FID is better for more diverse and realistic generated images.

## 5. Experimental Results

In this section, we show the effect of the feature merging technique on the quality of the generated image (Section 5.1), the effect of the number of labels, and training samples on the quality of the generated images (Section 5.2), a comparison between the proposed GAN models (Section 5.3) and the training stability of the proposed GAN models (Section 5.4), and comparisons with SOTA methods in semantic image synthesis (Section 5.5).

### 5.1. Effect of the Feature Merging Technique on the Quality of the Generated Image

**Figure 3:**
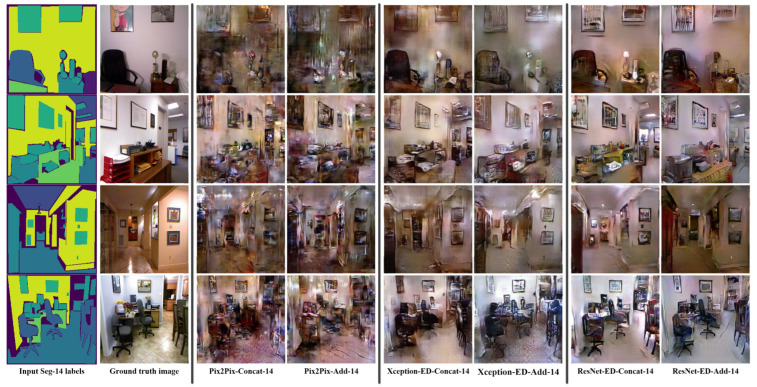
Sample results were obtained by Pix2Pix, Xception-ED, and ResNet-ED with concatenation and addition as feature merging of the encoder and decoder features while training with 14 semantic labels on the NYU depthV2 dataset.

**Figure 4:**
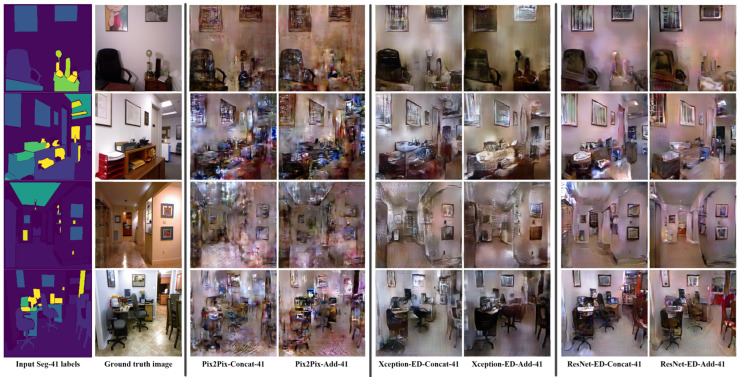
Sample results were obtained by Pix2Pix, Xception-ED, and ResNet-ED with concatenation and addition as feature merging of the encoder and decoder features, while training with 41 semantic labels on the NYU depthV2 dataset.

**Figure 5:**
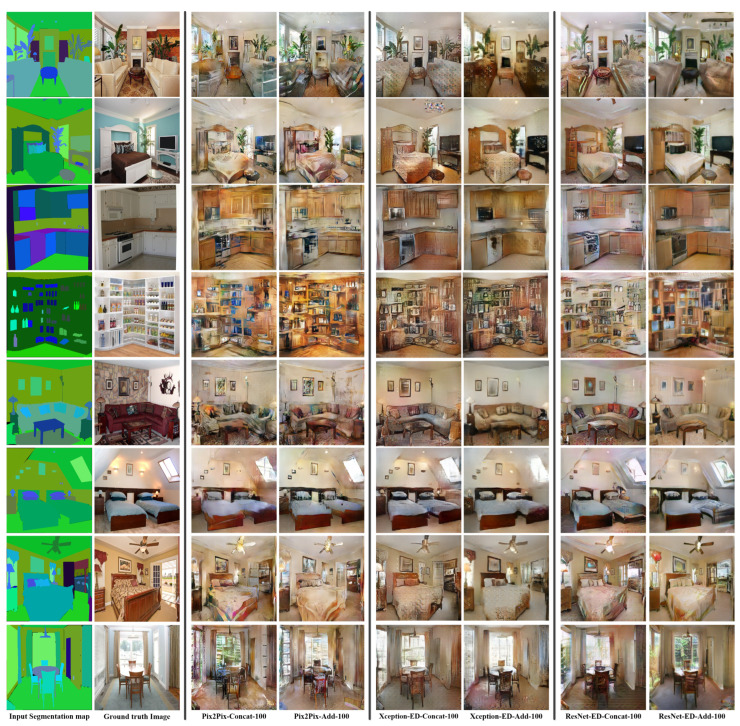
Sample results were obtained by Pix2Pix, Xception-ED, and ResNet-ED with concatenation and addition as feature merging of the encoder and decoder features while training with 100 semantic labels on the ADE20k indoor dataset.

We proposed merging the features of the same size in the encoder and decoder using concatenation and addition techniques. Merging by addition gives the model the ability to generate better quality images, and it consumes fewer computations as the number of parameters in case of addition is much less than that in the case of concatenation. Table 1 shows the number of parameters for each of the proposed architectures, highlighting that the number of parameters in the case of merging by addition is much less than that for the concatenation, while for the LPIPS (the best is 0.514 versus 0.505 for Pix2Pix with addition and with concatenation, respectively) and FID (the best is 122.732 versus 135.447 for ResNet-ED with addition and concatenation, respectively), the values are better, as well as in case of addition for architecture. Table 2 and Table 3 introduce better results for the case of merging by addition compared to merging by concatenation, proving that merging by addition is much more efficient and faster than merging by concatenation. Figure 3, Figure 4 and Figure 5 also show better quality images in the case of merging by addition for each architecture (Pix2Pix, Xception-ED, and ResNet-ED), whereas Xception-ED+Add generates better quality images than Pix2Pix+Add, and ResNet-ED+Add generates the best quality images of all.

### 5.2. Effect of the Number of Labels and Training Samples on the Quality of the Generated Image

We trained the proposed GAN models on the NYU-depthV2 dataset using a different number of labels. The original segmentation labels of the NYU depth V2 dataset were 14 labels, and the extended labels were 41 labels. Figure 3 shows the obtained results using the proposed models trained using 14 semantic labels, where Pix2Pix generates the worst quality, Xception-ED generates better quality images, and ResNet-ED generates the best quality images. Figure 4 shows the results obtained when the proposed models are trained on 41 semantic labels. The results, in this case, had better quality than the results in the case of 14 semantic labels. The proposed GAN models could learn the same object in the case of training on 41 semantic labels since each specific item or object had a unique label. In comparison, in the case of 14 labels, the labels were general. One label represented more than one object, so the GAN model generated general objects with many possibilities instead of the same target object. In the case of the ADE20K indoor subset, the proposed GAN models were trained on 100 labels, and the number of training images was 5991, which was much higher than that for NYU-depthV2 (1000 images). The results obtained using the model trained on ADE20K were much better in quality due to the higher number of labels (each specific object has a unique label) and training images than in the case of the NYU depth V2 dataset. Figure 5 shows the generated images using the proposed GAN models trained on the ADE20K indoor subset with 100 segmentation labels. Table 1, Table 2 and Table 3 also prove that the more semantic labels and data samples during training, the higher the quality of the generated images. The LPIPS and FID values in the case of the ADE20K indoor subset were much better (e.g., FID value of 81.067 for ResNet-ED+Add) than those for NYU depth V2 (e.g., FID values of 115.235 and 122.732 for ResNet-ED+Add with 41 and 14 semantic labels, respectively).

### 5.3. Comparison between the Proposed GAN Models

We compared the proposed GAN models to identify the best models in terms of LPIPS and FID metrics. In each comparison, in Table 1, Table 2 and Table 3, the best model in terms of FID metic was clearly ResNet-ED+ADD as it attained FID values of 122.732, 115.235, and 81.067 for 14 and 41 semantic labels of NYU depth V2 and 100 labels of the ADE20K subset. The FID values decreased, going down in the table from Pix2Pix to Xception-ED and then ResNet-ED, where merging by addition always has lower FID values than merging by concatenation. In the case of the LPIPS metric, ResNet was not the best in all cases, only for the NYU depth V2 with 14 labels. In the case of NYU depth V2 with 41 semantic labels and ADE20K subset with 100 semantic labels, Pix2Pix+Add had the highest LPIPS, which meant more diversity in the generated images; on the other hand, it had lower quality than the images generated by Xception-ED and ResNet-ED. The reason for that may be a bad quality itself of the generated images from Pix2Pix+Add, which make the images more diverse and lower in quality, but ResNet-ED+Add can still attain an acceptable LPIPS, which puts it at the top of the models with the best FID and an average LPIPS value.

### 5.4. Training Stability and Loss Improvement of the Proposed GAN Models

**Figure 6:**
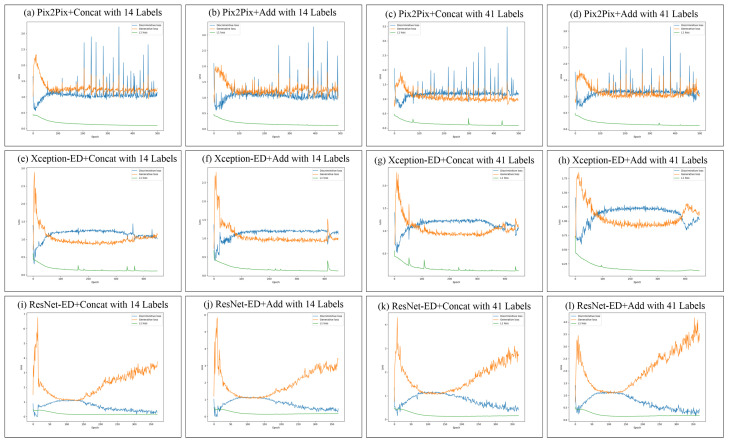
Loss improvement during training on NYU depth V2 dataset for Pix2Pix in (**a**–**d**), Xception-ED in (**e**–**h**), and ResNet-ED in (**i**–**l**) with concatenation and addition as feature merging of the encoder and decoder features with 14 and 41 semantic labels separately.

**Figure 7:**
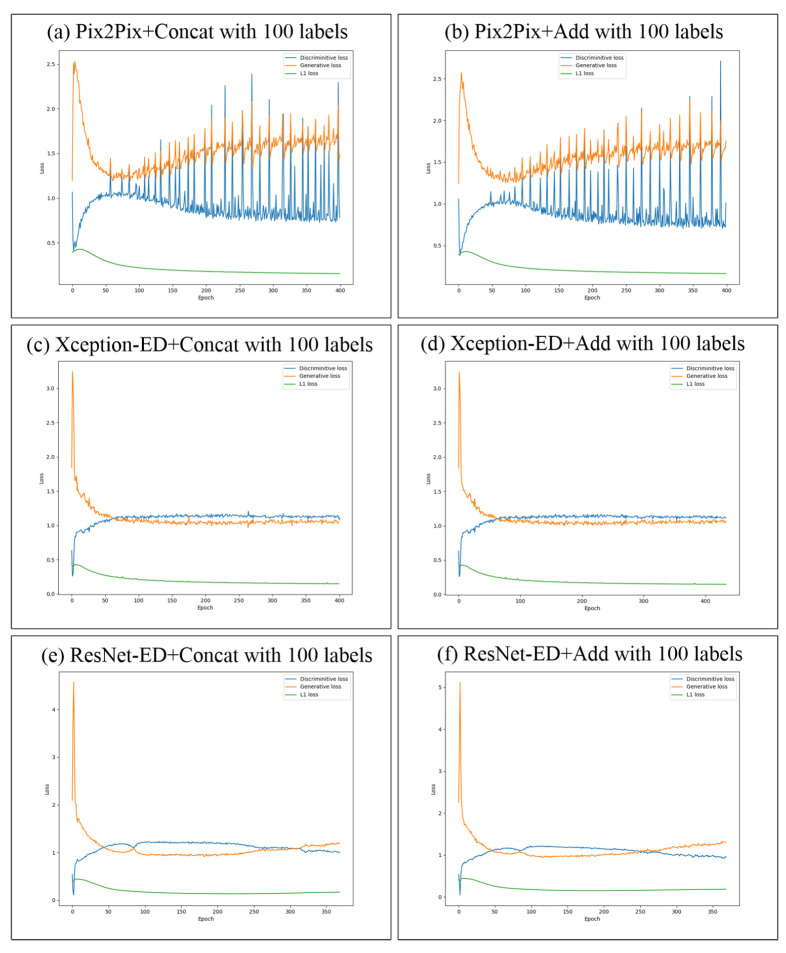
Loss improvement during training on ADE20K indoor subset for Pix2Pix in (**a**,**b**), Xception-ED in (**c**,**d**), and ResNet-ED in (**e**,**f**) with concatenation and addition as feature merging of the encoder and decoder features with 100 semantic labels.

In Figure 6, we show the loss improvement (the generator, discriminator, and L1 losses) of the proposed GAN models trained on NYU depth V2 with 14 and 41 semantic labels. While training Pix2Pix+Concat and Pix2Pix+Add on NYU depth V2, the training exhibited some overshooting, but still, the training was stable, and the generator and discriminator losses were converging. While training Xception-ED (with concatenation or with addition) on NYU depth V2, the training process was much more stable, and the losses smoothly improved with less convergence than in the case of the 14 labels. While training ResNet-ED (with concatenation or with addition), the training was stable, and the losses converged until epoch 170; after this epoch, the losses started to diverge, and the training process became less stable, so we tested the model in epochs 170∼180 to get the one that generates the best results. In the case of the proposed models that are trained on ADE20K indoor subset with 100 semantic labels (Figure 7), Pix2Pix-based models had very low stability during training, and the loss strongly fluctuated; however, in the case of Xception-ED and ResNet-Ed-based models, the training was extremely stable and smooth and the losses converged rapidly. In general, The images generated in the case of 100 labels of ADE20K were more realistic than those trained using 14 or 41 labels on NYU-depthv2, which was reflected in the stability and the convergence of losses during training. The number of labels and the dataset size plays important roles in the stability of the training process, as it is obvious from the visual comparison between the sub-figures in Figure 6 and Figure 7.

### 5.5. Comparison between the Proposed Method and State-of-the-Art Methods

**Figure 8:**
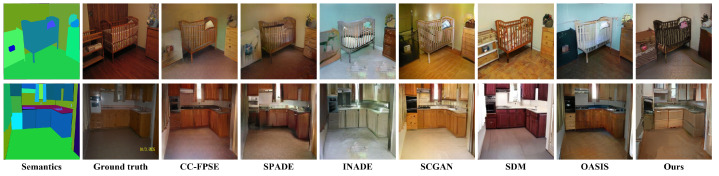
Quality comparison between the best proposed model (ResNet-ED+Add) and SOTA methods in image synthesis on ADE20K indoor subset; the methods in the comparison are CC-FPSE [27], SPADE [10], INADE [11], SCGAN [24], SDM [14], and OASIS [12].

We compare the best-proposed generator model (ResNet-ED+Add) with the SOTA methods in the semantic image synthesis task on the ADE20K Indoor subset. Table 4 shows the LPIPS and FID values obtained while validating those pre-trained models (pre-trained on ADE20K whole dataset) on the ADE20K indoor subset. The SOTA methods in the comparison are very recent methods in semantic image synthesis, published in the last 4 years (2018∼2022) including Pix2PixHd [9], SPADE [10], SCGAN [24], INADE [11], OASIS [12], and SDM [14] (which is the state-of-the-art best model published earlier in 2022 based on the diffusion models). The proposed model (ResNet-ED+Add) could attain the best (lower) FID (81.067) among other methods in comparison while it ranked two in the LPIPS as SDM [14] can attain the highest (best) LPIPS value, which means it has better diversity than the proposed model. Figure 8 shows a quality comparison between the proposed model (ResNet-ED+Add) and the SOTA methods on the ADE20K indoor subset. All of the methods in the comparison and our model can generate realistic images with extremely high quality to the point that one cannot tell that they are fake generated images as they have the same realism as the ground truth image, but with different styles and colors. The difference in the quality can only be sensed numerically from the comparison in Table 4, in which our method can attain the best FID value; however, this value is very close to SDM, OASIS, and SCGAN, which also attain very small FID values.

As the ADE20k indoor subset was custom-made in our study and never used in any previous research, testing in our selected test set gives the models trained on the ADE20K whole dataset an advantage. Our method was trained on specific labels, which were the indoor object labels that are supposed to give more concise output. In contrast, other methods in the comparison in Table 4 were trained on more objects (a total of 150 labels) and more training samples, which gives them the ability to generate more diverse objects so they can obtain better results (SDM attained better LPIPS value than ours) as the LPIPS and FID metrics measure both consistency and diversity (the generated output is better when it is consistent with the target image in terms of the image content and the location of each object, but it should be much different in style and colors than the ground truth image). SDM [14] seems to be better in LPIPS value than ours because the model was trained on more semantic labels and dataset size, so it might generate more diverse outputs than ours, which was trained on fewer labels and less datasets. Additionally, diffusion-based models naturally produce more variable outputs than GAN-based models in the image synthesis task, as proved in [28]. Therefore, we consider that attaining a better FID value than SDM is a good achievement of our study.

## 6. Future Work

In the current work, we achieved good quality of the generated images; however, in our future work, we will consider the deep unsupervised active learning technique [29] to improve the quality of the generated images, in which the model can learn and acquire new knowledge during the testing process. The well-predicted pixels or semantics during the test process can improve the model’s general knowledge so the overall accuracy can be improved.

## 7. Conclusions

The proposed GAN models could successfully construct artificial indoor images with high quality and impressive visual details, while input is a semantic label. It also generates better-quality images than Pix2Pix, which is proved in the experimental results section. The proposed merging with the Addition-based model is less complex than the UNet-based generator of Pix2Pix. We also proved by experiments that using more training images and semantic segmentation labels in the input segmentation map generates better quality images than the generic few labels. We also proved that the proposed residual models based on Xception and ResNet architectures could attain better results than those based on non-residual generator architecture (such as UNet). Additionally, the training process is much more stable, and the losses converge faster than those of Pix2Pix-based models. The proposed models attained good LPIPS, and FID values as the best model (ResNet-ED+Add) can attain an FID value of 81.067 and LPIPS value of 0.505, which outperformed the SOTA methods in semantic image synthesis in terms of FID and ranked 2 in terms of LPIPS. The proposed method benefits many modern applications, such as VR/AR applications, Image editing, interior design, and neural style transfer.

## Figures and Tables

**Table 1:** Comparison between the proposed GAN models trained on NYU depth V2 with **14 semantic labels** in terms of the generator and discriminator parameter count (G params. and D Params.) LPIPS and FID metrics.

GAN Model	ED Merging Technique	G params.	D params.	LPIPS	FID
Pix2Pix	Concatenatation	54,423,811	2,768,385	0.505	237.440
Pix2Pix	Addition	39,085,315	2,768,385	0.514	228.568
Xception-ED	Concatenation	33,452,515	41,825	0.471	203.329
Xception-ED	Addition	25,600,483	41,825	0.495	198.594
ResNet-ED	Concatenation	62,620,675	828,865	0.490	135.447
ResNet-ED	Addition	51,274,755	828,865	**0.509**	**122.732**

**Table 2:** Comparison between the proposed GAN models trained on NYU depth V2 with **41 semantic labels** in terms of LPIPS and FID metrics. Note that the generator and discriminator parameter counts for each model are the same as those values reported in Table 1.

GAN Model	ED Merging Technique	LPIPS	FID
Pix2Pix	Concatenation	0.520	230.413
Pix2Pix	Addition	**0.522**	218.635
Xception-ED	Concatenation	0.472	193.458
Xception-ED	Addition	0.465	189.727
ResNet-ED	Concatenation	0.473	130.681
ResNet-ED	Addition	0.497	**115.235**

**Table 3:** Comparison between the proposed GAN models trained on ADE20K indoor subset with **100 semantic labels** in terms of LPIPS and FID metrics. Note that the generator and discriminator parameter counts for each model are the same as those values reported in Table 1.

Method	ED Merging Technique	LPIPS	FID
Pix2Pix	Concatenation	0.515	140.241
Pix2Pix	Addition	**0.525**	137.905
Xception-ED	Concatenation	0.508	135.501
Xception-ED	Addition	0.522	131.256
ResNet-ED	Concatenation	0.491	95.402
ResNet-ED	Addition	0.505	**81.067**

**Table 4:** Comparison between the proposed ResNet-ED and SOTA methods on the ADE20K Indoor subset in terms of LPIPS and FID metrics. Note that all other methods are trained on the whole ADE20k dataset, while ours is only trained on the ADEK20 Indoor subset. Note that the best values in terms of LPIPS and FID are bolded.

Method	LPIPS	FID
Pix2PixHD [9]	0.125	135.271
DAGAN [25]	0.344	90.105
SPADE [10]	0.221	106.401
SCGAN [24]	0.341	87.141
GroupDNet [26]	0.232	117.511
CC-FPSE [27]	0.311	97.452
INADE [11]	0.491	109.340
OASIS [12]	0.364	85.322
SDM [14]	**0.552**	83.244
ResNet-ED+Add (**Ours)**	0.505	**81.067**

## Data Availability

The datasets used in this paper are public datasets. We provide the training code of the proposed method at: https://github.com/HatemHosam/Semantic-Label-Decomposition, accessed on 16 September 2022.

## Abbreviations

The following abbreviations are used in this manuscript:

GAN	Generative Adversarial Network
DCGAN	Deep Convolutional Generative Adversarial Network
CGAN	Conditional Generative Adversarial Network
Pix2Pix	image-to-image translation-based GenerativeAdversarial Network
CycleGAN	Cycle Consistency Generative Adversarial Network
StyleGAN	Style-Based Generator for Generative Adversarial Networks
SPADE	Spatially Adaptive Normalization
DAGAN	Dual Attention Generative Adversarial Network
SCGAN	Semantic Composition Generative Adversarial Network
INADE	INstance-Adaptive DEnormalization
OASIS	Only Adversarial Supervision for Semantic Image Synthesis
SDM	Semantic Diffusion Model
ED	Encoder-Decoder
Concat.	Concatenation-based architecure
Add.	Addition-based architecure
